# Dataset of tooth size measurements from the deciduous dentitions of 52 Spanish children. A reference collection for science

**DOI:** 10.1016/j.dib.2025.112342

**Published:** 2025-11-29

**Authors:** Marina Martínez de Pinillos, Chitina Moreno-Torres, Leslea J. Hlusko

**Affiliations:** Centro Nacional de Investigación sobre la Evolución Humana (CENIEH), Paseo Sierra de Atapuerca 3, 09002 Burgos, Spain

**Keywords:** Ratón Pérez collection, Deciduous teeth, Mesiodistal diameter, Bucolingual diameter, Crown index, Crown area, modern *Homo sapiens*

## Abstract

This article presents a dataset of mesiodistal and buccolingual crown measurements from 712 deciduous incisors, canines, and molars belonging to 52 Spanish children from the Ratón Pérez Collection. This collection housed at the National Research Center on Human Evolution (CENIEH, for its acronym in Spanish) in Burgos, Spain, constitutes one of the most valuable citizen science initiatives on the international stage. The dataset offered in this article is unusual and especially valuable because it belongs to individuals who have donated at least ten associated teeth over the years. Measurements were obtained following standardized odontometric protocols to ensure accuracy and comparability. Unlike other studies, here we provide all individual measurements, making it the first openly available reference collection of raw deciduous tooth metrics derived from a well-documented and ethically-sourced sample. These data enable exploration of variability in primary tooth size, facilitate comparisons with other populations, and support studies on growth, development, and child forensic identification. By making this original dataset publicly available, we contribute a valuable tool for multiple scientific disciplines such as pediatric dentistry, forensic sciences, bioarchaeology, dental anthropology or human evolution among other disciplines.

Specifications Table

**Table:** 

Subject	Social Sciences
Specific subject area	Dental anthropology: linear dimensions of the mesiodistal and buccolingual diameters of deciduous incisors, canines and molars using a digital caliper.
Type of data	Table
Data collection	The dataset contains the mesiodistal (MD) and buccolingual (BL) diameters, crown index (BL/MDx100) and crown area (BLxMD) of deciduous incisors, canines and molars from the Ratón Pérez collection. We gathered the dental measurements of each tooth at the level of the maximum crown diameter. These two dimensions of the crowns were taken only once by the same person with a Mitutoyo digital sliding caliper and recorded to the nearest 0.1 mm following the method outlined by [1]. The 712 teeth offered in this study belong to 52 children from eleven different cities in Spain who, between 2014 and 2022, donated at least ten teeth to the collection and whose ages at tooth loss range from 3 to 15 years.. Teeth with severe attrition or lesions affecting the contour of the crown were not measured.
Data source location	Centro Nacional de Investigación sobre la Evolución Humana (CENIEH), Paseo Sierra de Atapuerca 3, 09,002 Burgos, Spain.
Data accessibility	Repository name: Zenodo https://zenodo.org Data identification number: 10.5281/zenodo.17255974 Direct URL to data: https://doi.org/10.5281/zenodo.17255974
Related research article	Martínez de Pinillos, M., Pantoja‐Pérez, A., Fernández‐Colón, P., Martín‐Francés, L., García‐Campos, C., Modesto‐Mata, M., … Martinón‐Torres, M. (2021). The Ratón Pérez collection: Modern deciduous human teeth at the Centro Nacional de Investigación sobre la Evolución Humana (Burgos, Spain). American Journal of Physical Anthropology, 176, 528–535. https://doi.org/10.1002/ajpa.24371

## 1 Value of the Data

• **Unique reference dataset of deciduous dentition:** these data comprise 712 individual measurements (mesiodistal, buccolingual, crown index, and crown area) from 52 Spanish children, who have donated at least 10 teeth over the years. This makes it one of the most complete and well-documented modern reference datasets of deciduous dentition. By making this original dataset publicly available, we contribute a valuable tool for multiple scientific disciplines such as pediatric dentistry, forensic sciences, bioarchaeology, dental anthropology, and human evolution, among others.
• **High reusability through standardized methodology:** measurements were obtained following established odontometric protocols [1], ensuring precision, reproducibility, and comparability with other datasets. Researchers can reuse these data for morphometric, developmental, and comparative analyses, as well as integrate them into broader quantitative studies on human dental variation in both modern and fossil samples.
• **Comparative framework for evolutionary and anthropological studies:** the dataset provides essential baseline information for interpreting morphological variability in fossil deciduous teeth. It supports analyses of taxonomic differentiation, developmental trajectories, and evolutionary patterns within the genus *Homo*, enabling more robust comparisons between modern and ancient populations.
• **Applications in pediatric dentistry and forensic sciences:** these data can be reused to establish normative standards for dental growth and development, helping to detect developmental anomalies or health-related dental conditions in children. In forensic contexts, the dataset facilitates age estimation and biological profiling of subadult individuals, contributing valuable reference data where modern collections are scarce.
• **Ethically curated and richly contextualized resource:** derived from the Ratón Pérez Collection, this dataset is ethically sourced, anonymized, and accompanied by detailed contextual information (age, sex, ancestry, feeding type, gestational length). Its rigorous curation and interdisciplinary value make it a model resource for biomedical, anthropological, and evolutionary research.

## 2 Background

The motivation behind compiling this dataset stems from the increasing recognition of deciduous teeth as valuable biological archives across multiple scientific disciplines, including anthropology, paleontology, dentistry, and forensic science. Deciduous dentition preserves a wealth of morphological, genetic, and chemical information that contributes to understanding human development, health, and evolutionary history. Despite their diagnostic potential, modern reference collections of deciduous teeth with complete biographical documentation remain scarce worldwide.

**Fig. 1:**
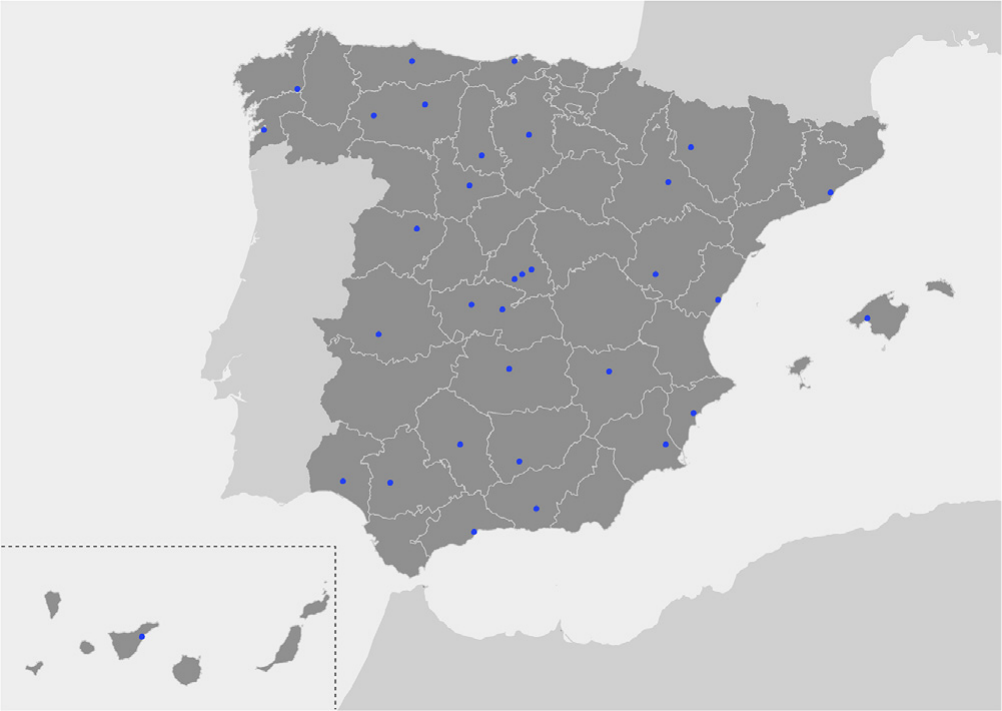
Tooth collection sites throughout Spain during the different campaigns from 2014 to 2025.

Against this backdrop, the Ratón Pérez Collection, established in 2014 at the CENIEH in Burgos (Spain) was conceived as a citizen science initiative to fill this gap (Fig. 1). It integrates modern deciduous teeth with associated contextual information such as age, sex, ancestry, and developmental history (see Fig. 1 of Supplementary Material). The present dataset specifically provides metric data on the mesiodistal and buccolingual diameters of all deciduous teeth measured following standardized protocols. These measurements are widely used in human evolution (for taxonomic and phylogenetic purposes), forensic applications (for identification and age estimation), and pediatric dentistry (for diagnostic and developmental studies), among other disciplines.

The data article expands on previous research [[2], [3], [4], [5]–6] by describing the dataset’s structure, documentation, and ethical framework, thereby enhancing reproducibility and facilitating its use in comparative, biomedical, and evolutionary contexts.

## 3 Data Description

**Fig. 2:**
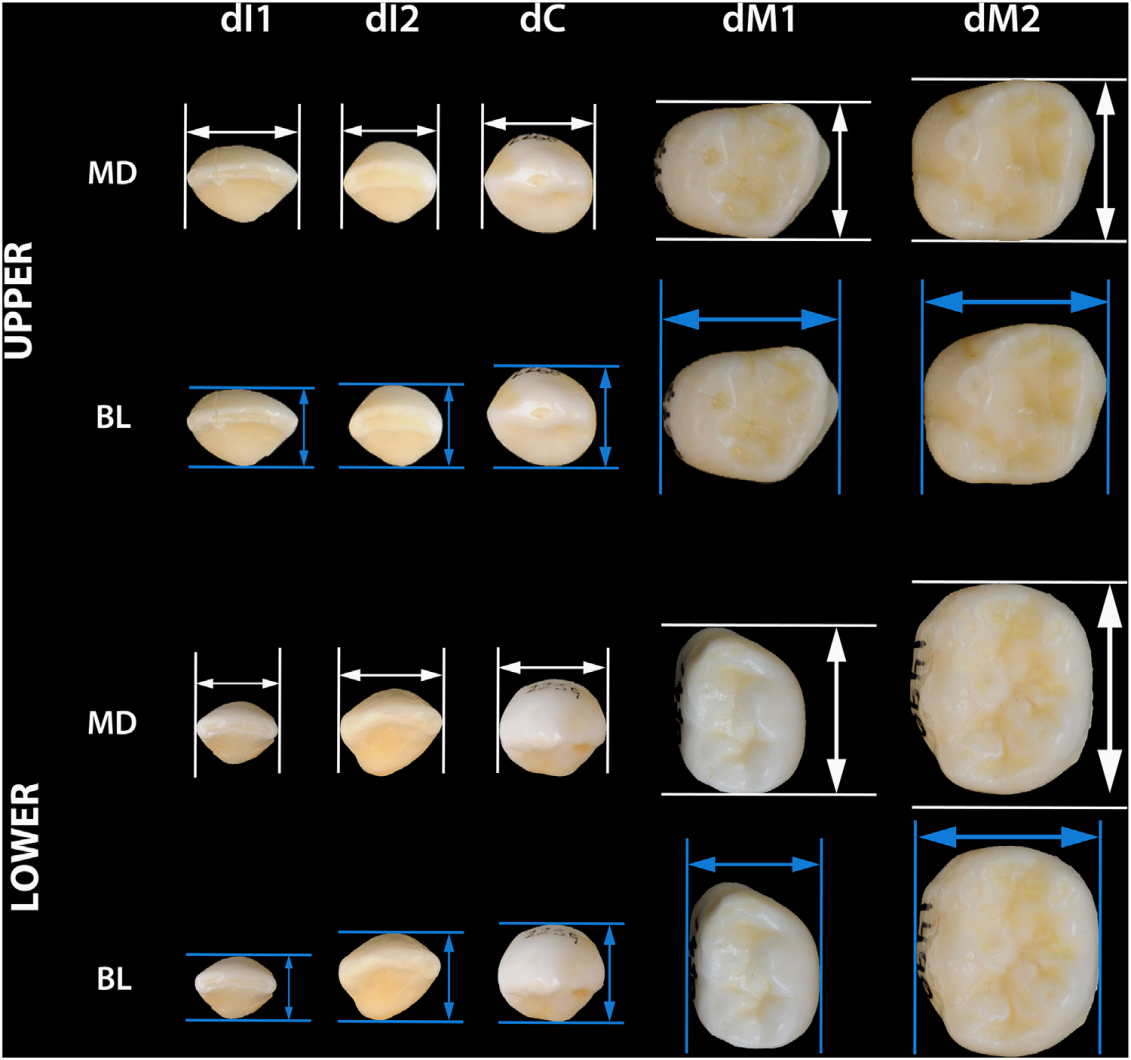
Location of measurements on the upper and lower deciduous dentition (teeth not to scale). The white arrows represent the maximum mesiodistal diameters (MD), and the blue arrows represent the maximum buccolingual diameters (BL).

The dataset contains the mesiodistal and buccolingual diameters, crown index (BL/MDx100) and crown area (BLxMD) of deciduous incisors, canines and molars from the Ratón Pérez collection. We gathered the dental measurements of each tooth at the level of the maximum crown diameter (Fig. 2). These two dimensions of the crowns were taken with a Mitutoyo digital sliding caliper and recorded to the nearest 0.1 mm following the method outlined by [1]. For this study, the tooth measurements were taken only once by the same person using the same digital sliding caliper each time. The 712 teeth offered in this study belong to 52 children from eleven different cities in Spain who, between 2014 and 2022, donated at least ten teeth to the collection and whose ages at tooth loss range from 3 to 15 years. Teeth with severe attrition or lesions affecting the contour of the crown were not measured.

The data spreadsheet is organized and ordered by donors and the abbreviations are as follows:

**Table:** 

Tooth ID*	Number that corresponds to the donated tooth	
Individual ID	Number that corresponds to the donor	
Dimensions	MD	mesiodistal
	BL	buccolingual
	CI	crown index
	CA	crown area
Sex	M	male
	F	female
Tooth and position	dI	deciduous incisor
	dC	deciduous canine
	dM	deciduous molar
	1	first
	2	second
Jaw	U	upper
	L	lower
Side	R	right
	L	left

* The six teeth marked with an asterisk have small fillings on the mesial or distal surfaces, but these fillings do not interfere with the measurement of the mesiodistal and buccolingual diameters.

## 4 Experimental Design, Materials and Methods

The Ratón Pérez Collection was established in 2014 through the voluntary donation of naturally shed deciduous teeth from children across Spain [6]. Collection procedures followed standardized biomedical and paleoanthropological protocols identical to those used for fossil specimens at the National Research Center on Human Evolution (CENIEH, Burgos, Spain).

First, for the initial cleaning, the teeth are gently washed using a 1:1 solution of purified water and ethanol to eliminate any adhered organic residues. Once dry, each tooth is stabilized with a diluted resin solution (3 % Acryloid B72 in acetone) to strengthen it and ensure proper preservation. Every tooth receives an inventory code and is stored individually in a zip-lock bag labelled accordingly. The label specifies the tooth’s inventory number, the donor’s identification code, the tooth type, the individual’s sex, the age at tooth loss, the number of the storage box, and a checkbox indicating whether the tooth has been scanned. Between 50 and 75 individually bagged teeth are placed in transparent polystyrene boxes, which are then stored inside polypropylene containers.

For each sample, parents or legal representatives completed and signed a detailed form including the child’s age, sex, tooth position, date of exfoliation, geographical origin, parental ancestry, gestational length, type of infant feeding, and relevant medical information. The teeth and the data collected for this study are ethically sourced, with full compliance with current legislation and bioethical standards. All samples are identified only with an alphanumeric code, ensuring that the donor’s identity will never be revealed. Access to personal data is strictly limited to the person responsible for the research team, and only when necessary to verify the data and study procedures. At all times, this information is kept in the strictest confidence, in accordance with Act 14/2007 on Biomedical Research and complementary regulations, and under the approval of the Bioethics Commission of the University of Burgos.

Metric data were obtained by recording the maximum mesiodistal (MD) and buccolingual (BL) diameters of deciduous incisors, canines, and molars. The mesiodistal diameter was measured as the maximum distance between the contact points on the mesial and distal surfaces, while the buccolingual diameter was measured as the maximum distance between the buccal and lingual surfaces, perpendicular to the mesiodistal axis. Measurements were taken using a Mitutoyo digital sliding caliper and recorded to the nearest 0.1 mm following the method outlined by [1].

## Limitations

These dental measurements were derived from deciduous teeth that were voluntarily donated for scientific research led by the Spanish National Research Center on Human Evolution. As can be seen in the data spreadsheet, most donors came from Burgos, although there were also donors from 10 other cities in Spain. While the community of Burgos, were the vast majority of donations were received, is a fairly homogeneous population of white Europeans, it is possible that there is some socioeconomic bias due to the voluntary nature of the collection process. However, we note that the Burgos community has an unusually high level of engagement with the science of human evolution given the proximity to the site of Atapuerca and the national pride taken in this history. Although socioeconomic data were not collected alongside the teeth, from the conversations had at the time of donation, anecdotally, the collection is fairly representative of the Burgos community broadly.

## Ethics Statement

This collection is currently placed at the National Research Center on Human Evolution (CENIEH) in Burgos, Spain. All the data contained therein are anonymous in accordance with Spanish Law (Act 14/2007, of July 3rd, Chapter III, Article 20, on Biomedical Research) and has been approved by the Bioethics Commission of the University of Burgos.

## Credit Author Statement

**Marina Martínez de Pinillos:** Conceptualization, Collection manager, Data curation, Formal analysis, Investigation, Methodology, Writing- Original draft, Review and Editing. **Chitina Moreno-Torres:** Collection manager, Funding acquisition, Project administration, Resources. **Leslea Hlusko:** Conceptualization, Collection manager, Formal analysis, Investigation, Methodology, Writing- Original draft, Review and Editing.

## Data Availability

ZenodoTooth size measurements from the deciduous dentitions of 52 Spanish children. (Original data)

## Appendix Supplementary materials

Image, application 1
