# Supplementary material for: Dataset of tooth size measurements from the deciduous dentitions of 52 Spanish children. A reference collection for science
Source: Data Brief. 2025 Nov 29;64:112342. doi: 10.1016/j.dib.2025.112342 (PMC12756538; doi:10.1016/j.dib.2025.112342)
Supplement: Supplementary file 1 [file mmc1.pdf]

DATA SHEET – DECIDUOUS TOOTH COLLECTION CAMPAIGN (RP20____- GAD)

☐ The donor has provided teeth in the past.
☐ Extraction

CENIEH-22/ INDIVIDUAL Nº ______________	
**Name and surname**	
**Date and place of birth** (dd/mm/yyyy)	......./........./............. in.. Country: ....
**Normal place of residence**	In... Country: ....
**Sex**	Female ☐ Male ☐
**Age of the donor at which tooth fell** (as accurate as possible)	

**BIRTH**	☐ Premature (earlier than 37 weeks of pregnancy or the eighth month)
	☐ On time (between weeks 37 and 41 of pregnancy or in the ninth month)
	☐ After term (after week 41 of pregnancy or the ninth month)
**NURSING**	☐ Breastfeeding
	☐ Bottle-feeding
	☐ Mixed feeding (breastfeeding + bottle-feeding)

ASCENDANTS

Relation	Place of birth	Normal place of residence
Father	In.................................. Country:	In... Country: ....
Mother	In................................... Country:	In... Country: ....
Paternal grandfather	In.................................. Country:	In... Country: ....
Paternal grandmother	In................................... Country:	In... Country: ....
Maternal grandfather	In................................... Country:	In... Country: ....
Maternal grandmother	In................................... Country:	In... Country: ....

REMARKS (other significant data you wish to provide)
